# On Some of the Diseases of the Testis

**Published:** 1830-10-01

**Authors:** 


### XLIII.

### M. LARREY ON SOME OF THE DISEASES OF THE TESTIS.*

THIS veteran, whose experience in the tented field has been great, and whose situation as surgeon in chief to the Military Hospital of the Royal Guard must have enhanced his opportunities for observation, has published in his Clinique Chirurgicale some interesting remarks on diseases of the testicle. Perhaps it may be instructive to compare the opinions of the worthy Baron with those of Sir Astley Cooper, on some points of pathology and practice.

1. *Wounds of the Testicle.* M. Larrey has remarked that these are not succeeded by such bad effects, as the nature and sensibility of the organ would have led us to suppose. A Swiss was lately in the Military Hospital, who had received a cut from a very sharp knife through the whole left side of the scrotum. The instrument had divided the tunica vaginalis, and corresponding portion of the testicle. The wound was dressed with mild ointment and compresses dipped in a camphorated wash, and the scrotum suspended; little suppuration took place; and on the twenty-fifth or twenty-sixth day the wound was perfectly healed. The testis appeared smaller and more contracted than its fellow. If the testis is so injured by a projectile as to be denuded of its tunics, or extensively destroyed

---

* Clinique Chirurgicale, Tome III.

in its texture, it must of course be removed. If a violent contusion is followed by ecchymosis, leeches, slight compression by dressings dipped in a stimulating lotion, as one of camphor or ammonia, position, diet, and a gentle emetic to prevent the sympathetic affection of the stomach, are the measures employed by M. Larrey with success.

2. *Inflammation of the Testicle.* This may be either from over exertion or sympathetic. The first is rather rare, and is the only one, according to M. Larrey, for which leeches ought to be applied. After these, discutient and sedative lotions with gradual compression effect a cure. The swelling of the organ occasioned by extreme continence and retention of the seminal secretion is characterized by the size of the part, the tensive pain, the rapid dilatation of the spermatic veins, and the great inconvenience experienced in walking. Baths of cold water, even ice, cooling drinks, and the horizontal posture speedily relieve the symptoms. M. Larrey denies that great continence is so injurious as some authors have supposed.

In the sympathetic inflammation of the testis from gonorrhœa, the membranes are affected as well as the gland itself; if left to itself it may terminate in abscess, but rarely in sloughing. Sanguineous evacuations or leeches are, in M. Larrey's opinion, more injurious than beneficial, and seem to give rise to abscess in some, to hydrocele in others. We doubt whether many practitioners on this side of the Channel will agree with the Baron on these points. His practice consists in introducing into the urethra a small elastic bougie, spread thickly with a gummy preparation of opium, and in giving sedative demulcent drinks, with pills of camphor or of nitre and hyosciamus. An embrocation of camphorated oil of camomile is rubbed upon the scrotum, and slight compression applied by means of a flannel suspensory. When resolution is commencing a gentle emetic is prescribed. This mode of proceeding is hardly to be compared with the more energetic one adopted in this country.

When suppuration takes place, which is usually in the epididymis, we should encourage it by fomentations, and open it as early as fluctuation can be felt. The abscess generally heals without difficulty, excepting when the body of the testis is affected; this is usually deeply disorganized and destroyed.

3. *Nervous Affection, or "Irritable Testis."* M. Larrey has only seen two cases of this kind, one in an officer of the guard, and the other in a young Parisian lawyer. It was characterised by violent pain extending from the cord to the testicle and occurring in variable paroxysms, retraction of the testis during the latter, moroseness and despondency of temper, and loss of sleep. M. Larrey has remarked, as Sir Astley Cooper has done, that depletion is injurious. If the pain attacks the loins, M. L. uses cupping and moxa, and the latter may be applied in the course of the cord. These means succeeded perfectly in the two cases already adverted to.

4. *Wasting of the Testicle.* This has been remarked by Sir Astley Cooper as a consequence of inflammation, and by Mr. Brodie, if we remember right, of indulgence in masturbation. Baron Larrey gives a more detailed account of the several causes of this curious affection. Sometimes, when the swelling produced by mechanical injury has subsided, the testicle gradually diminishes in size until it completely wastes. In some cases which our author relates in another part of the work, a wound in the back of the neck, affecting the cerebellum, has been followed by more or less wasting of these organs. The abuse of venery; the employment of preparations of opium, whether applied externally or injected into the urethra for gonorrhœa; and especially immoderate indulgence in alcoholic liquors containing much narcotic matter, are very active causes of the complaint.

At the end of the first campaign in Egypt, a number of the soldiers of the French army complained of the almost total disappearance of their testicles, without any venereal affection to account for it. They remarked that they began by losing the sensibility of the generative organs, which no longer preserved their vigour or their form, but gradually softened. So slow and insensible was the change, that they usually only discovered the malady when the testicles had nearly disappeared. On examination at this period, they were found near the ring resembling beans, whilst the cord was equally diminished and wasted. When both testicles were affected, the patient was deprived of his sexual powers and desires; he became melancholic ; the voice was altered ; and the beard ceased to grow. Nearly fifty soldiers were judged incapable of service on these accounts.

M. Larrey attributes the disease to the extreme heat of the Egyptian climate and the laborious marches through the desert, which softened the texture of the testicle, and occasioned at first a kind of enlargement, succeeded by the wasting in question. M. Larrey also assigns a destructive effect to the use of alcoholic and narcotic substances, but cannot explain very clearly their modus operandi. Into the composition of the brandy of the country, made from dates, there enter several plants of the class of solanum, such as the pimento and the berries of the cherry laurel. M. Larrey thinks it probable, that the action which such substances exercise on the nerves of the stomach, is transmitted sympathetically to the testicles, and occasions their absorption. The ancients, it is said, procured the same thing by the application, for a length of time, of the concrete juice of hemlock to the scrotum. These conjectures of the Baron's must be taken for what they are worth, but it is not improbable that the immoderate use of such substances, combined with fatigues in a burning and enervating climate, may exercise a mysterious agency on the glands of the testes.

When the wasting is complete, art possesses no power to renovate the organ. In the earlier stages of the malady, we may, perhaps, effect some benefit by withdrawing, as far as possible, its causes, and by employing some vapour-baths, with dry friction on the surface of the body, irritation in the lumbar and sacral regions, tonics, and generous food. Spirituous liquors should be avoided, or, at all events, procured without adulteration. A suspensory ought always to be worn in warm climates, and frequent ablutions of the body with cold vinegar and water, and abstinence from immoderate venery, are necessary as preventive measures. M. Larrey has had several soldiers affected with this complaint under his care in France. It pursued the same course as in Egypt, and the patients confessed that they had been addicted to immoderate indulgence in venery, and strong, adulterated spirituous liquors. In one of these individuals, both testes in a short time almost disappeared. From being originally of a very robust constitution, he lost his beard and manly features, and looks like a woman. A soldier, whilst landing from a vessel in Egypt, received a violent blow upon the back of the neck, after which the testicle wasted to the utmost degree. These facts collected by the Baron are curious and worth perusal.

5. *Hypertrophy of the Testicles.* An excessive growth of the female breast, without any appreciable change of structure, is not a very uncommon circumstance, but we were not previously aware of the existence of such an affection of the testicle. M. Larrey has seen it twice in the Hospital of the Guard. The first patient was 26 years of age, and both the testicles had acquired considerable volume, without exhibiting any perceptible morbid alteration, or occasioning inconvenience, except from their weight. The penis was incapable of erection, but the general health was good. Frictions on the scrotum and in the course of the cord with "double Neapolitan ointment," in small quantities and at long intervals ; embrocations of camphorated oil of camomile; uniform compression by a flannel suspensory ; slight diaphoretics, combined with bitters, and the horizontal position, reduced the testes in three months to their natural size. In the other case, the testicles were as large as the fist, and, although in excellent health, this patient also had lost the power of erecting the penis. The same treatment as before, continued for six months, was perfectly successful.

